# Inequalities in geographical distribution of heritage in Scotland, investigating spatial exposure to heritage sites through area-based and individual-based (GPS) measurement

**DOI:** 10.1016/j.wss.2024.100211

**Published:** 2024-12

**Authors:** Laura Macdonald, Fiona Caryl, Richard Mitchell

**Affiliations:** MRC/CSO Social and Public Health Sciences Unit, University of Glasgow, Clarice Pears Building, 90 Byres Road, UK

**Keywords:** Heritage, Children, Deprivation, Inequalities, Geographic information system, Global positioning system

## Abstract

Growing evidence suggests that visiting heritage, such as historic buildings, or landscapes, may enhance mental health. However, adults and children from deprived areas are less likely to visit heritage, perhaps partly due to unequal geographical availability. Few studies investigate socio-spatial inequity of heritage access nationally, and there is an absence of research exploring individual-based contact with heritage. We study both and ours is the first to use Global Positioning System (GPS) data to examine the latter. For Scotland we quantify area-level ‘opportunities’ for heritage contact (i.e., data zone-level counts of sites), and SPACES Study children's (*n* = 688) individual-level heritage ‘encounters’ (i.e., GPS buffer-level counts of sites), by income deprivation. Heritage exposure varied by deprivation at area- (ANOVA *p* < 0.001) and individual-level (*p* = 0.003); in poorer areas there were fewer ‘opportunities’ to visit heritage (mean numbers in most deprived: 2.8 (CI:2.2–3.5), and least deprived: 11.7 (CI:10.3–13.1)) and children had fewer ‘encounters’ with heritage (most deprived: 32.6 (CI:25.3–40.2), and least deprived: 58.0 (CI:47.9–69.3)). Inequalities at individual-level were smaller than at area-level; children in poorer areas appeared to compensate for fewer sites near home during their daily movements. Nonetheless, inequalities in exposure did persist, and opportunities to access heritage remained unequal to the detriment of those in poorer areas. Our evidence is policy relevant, highlighting a need for place-based schemes to address geographic inequalities in access. For example, targeted investment in heritage in/near deprived areas; free site entry for those on lower incomes; and educational outreach activities to improve awareness of local heritage.

## Highlights

• Lower heritage engagement in poorer areas may be due to unequal spatial access.
• We use a novel application of GPS to quantify heritage contact for 688 children.
• Poorer areas in Scotland had fewer opportunities to visit heritage sites locally.
• Children living in poorer areas had lower contact with heritage day-to-day.
• Place-based schemes are needed to address geographic inequity in heritage access.

## 1 Introduction

A growing evidence base suggests that visiting historical sites and engaging with heritage, can play a role in enhancing mental health and well-being in both adults and in children (Leadbetter and O'Connor, 2013; Brown et al., 2015, Lakey, Smith et al., 2017, Mak, Coulter et al., 2021, Macdonald, Nicholls et al., 2023). By heritage sites we mean protected buildings, structures, or landscapes that are officially recognized for their historical, cultural, or architectural value (e.g., listed buildings, prehistoric forts and monuments, medieval castles, stately homes, designed landscape, churches, war memorials etc.). Heritage site designations, and criteria for designation, vary from country to country and can differ at local, national, and international levels. Local landmarks may be designated by local authorities; national heritage by national bodies (e.g., Historic Environment Scotland, Historic England, or Cadw (Wales) in Great Britain); and world heritage by international organisations such as the United Nations Educational, Scientific and Cultural Organisation (UNESCO).

Research shows that adults who frequently engage with heritage benefit from higher levels of self-esteem, life satisfaction, and happiness, and lower levels of mental distress (Leadbetter and O'Connor, 2013; Brown, MacDonald et al., 2015, Lakey, Smith et al., 2017, Mak, Coulter et al., 2021). Residents who live in areas with greater numbers of heritage sites also experience greater feelings of belonging and place attachment, and a ‘sense of place’ has been associated with better life quality and mental health (Bradley, Coombes et al., 2009). A smaller evidence base explores the influence of heritage on children's well-being. It has been argued that children's sense of place can be positively influenced by local heritage, and place attachment is valuable for children as they grow into adulthood (Grimshaw and Mates, 2022). Sense of place can contribute to well-being and identity formation (Grimshaw and Mates, 2022), and can encourage engagement in community-led activities (Severcan, 2015). Furthermore, young people may acquire various well-being and behavioural benefits from involvement in heritage activities, and other sport and cultural pursuits (Lakey, Smith et al., 2017).

Despite the value of heritage engagement, in the UK, adults and young people from poorer households are less likely to visit heritage sites compared to their higher-income counterparts (Lakey, Smith et al., 2017, Lakey, Smith et al., 2017). This pattern is mirrored across Europe (Falk and Katz-Gerro, 2016) and beyond (Ateca-Amestoy, Gorostiaga et al., 2020). Although reasons for lack of engagement amongst disadvantaged families are complex, including a plethora of individual and socio-cultural factors, it may also be driven by inequality in geographical availability of heritage (Mak, Coulter et al., 2021). Recent research showed that the most deprived Lower Super Output Areas (LSOAs) (i.e., small area units created for collecting statistics about populations) in England had fewer heritage sites per population, and that LSOA-level availability of heritage was associated with visits to heritage (Macdonald, Nicholls et al., 2023). Within poorer areas, fewer opportunities to access heritage may impede heritage engagement.

In this study we seek to fill several research gaps. Firstly, our study is pertinent as there remains a scarcity of studies in any setting investigating socio-spatial inequity of access to ‘cultural amenities’, such as heritage (Moldavanova, Meloche et al., 2022). An understanding of geographical availability of heritage provides an opportunity to feed into place-based solutions to improve inequity, with governments acknowledging a need to do so (Scottish Government, 2020; HM Government, 2022). Secondly, there is no research to date exploring individual-based contact with heritage such as number/location of ‘encounters’ in every-day life. Existing surveys including questions on heritage visits (e.g., The Participation Survey, the UK Household Longitudinal Survey) ascertain frequency of visit and category of site but not the location of heritage contact (e.g., within local area or beyond). Thirdly, there are no studies measuring area- and individual-level heritage exposure and potential inequalities in both. Comparison of inequalities for both will give us greater understanding of whether individuals can mitigate any area-level inequality through their daily mobility. We focus on inequalities and quantify area-level ‘opportunities’ for local heritage contact, and individual-level heritage ‘encounters’. By ‘opportunities’ we mean counts of heritage sites located within small-area geographies (i.e., data zones), and by ‘encounters’ we mean counts of heritage sites passed by/through day-to-day by individuals. Our approach measures individual ‘encounters’ using existing mobility Global Positioning System (GPS) data collected across multiple days from ‘Studying Physical Activity in Children's Environments across Scotland’ (SPACES) (MRC/CSO SPHSU, 2023). GPS data use allows capture of the ‘dynamic nature’ of individual's daily contact with their environment (Kirchner, Cantrell et al., 2012), and such individual-based measures provide a better understanding of heritage exposure as they take account of day-to-day locations/routes beyond the extent of the home neighbourhood. Studies quantifying neighbourhood exposure using fixed area-level units (e.g., census areas), may under/over-estimate exposure (Caryl, Shortt et al., 2020), and divergence between area- and individual-level measures may prompt revaluation of conclusions drawn solely from the former. Interventions or policies based on such conclusions may prove ineffective or even exacerbate inequality.

We regard our research as opportune as heritage organisations seek to heighten interest and engagement in heritage, particularly in young people, and in underrepresented groups, such as those in deprived areas (Blamire, Rees et al., 2022) and governments prioritise the enhancement and preservation of heritage for future generations (Scottish Government, 2020; HM Government, 2022). Throughout the research process, we engaged with our Historic Environment Scotland collaborators to optimise generation of meaningful and policy-relevant research evidence. Our overarching aim is to understand the geographical availability of heritage more generally, individual encounters with heritage specifically, and inequalities therein.

Our main research question is ‘do area-level and individual-level measures of heritage exposure vary by income deprivation in Scotland?’, with a subsidiary question ‘do area-level and individual-level measures show similar levels of inequality?’. We posit that area- and individual-level findings indicate inequalities by deprivation (to the detriment of poorer areas), and either inequality is comparable for both, or individual-level inequality is reduced.

## 2 Methods

### 2.1 Area-level exposure to heritage (Scotland)

To calculate area-level exposure to heritage (i.e., ‘opportunities’) across Scotland we obtained/linked (1.1) heritage data and (1.2) deprivation data; (1.3) calculated area-level measures of heritage exposure and compared these measures across area-level income deprivation quintiles (see detail below).

#### 2.1.1 Heritage data

We obtained spatial data on heritage sites (*n* = 76,711), that is, Listed Buildings points, Scheduled Monument polygons, and Gardens and Designed Landscape polygons, for 2015, from the Scottish Government Spatial Data portal (Scottish Government, 2023) (data publicly available under the Open Government License). Historic Environment Scotland is the leading public body maintaining Scotland's historic environment, and responsible for listing buildings for the Scottish Government (HES, 2023). The dataset includes statutory addresses for Listed Buildings, that is buildings/structures of ‘special’ interest as acknowledged via the Planning (Listing Buildings and Conservation Areas) (Scotland) Act 1997. To provide a more accurate representation of the geographical extent of Listed Buildings, their points were linked to Ordnance Survey Master Map Topology building polygons (2015) (downloaded from (EDINA, 2023)). Around 80 % of points matched to polygons, while the non-matched 20 % remained as points and were non-building structures, such as bridges, fountains etc. (see Table 1 for examples). Scheduled monuments are nationally important structures/sites, legally protected by the Ancient Monuments and Archaeological Areas Act 1979. The Scottish Historic Environment policy sets out criteria to assess national importance accounting for various factors including artistic, architectural, historic, archaeological, aesthetic, scientific etc. Gardens and Designed Landscapes are grounds that have been created for artistic effect, with their criteria for national importance determined by Scottish Historic Environment Policy; the inventory is maintained by Historic Environment Scotland.

**Table 1:** Heritage – additional information.

**Heritage vector data type (n)**	**Examples of sites**
Listed buildings as polygons* (56,311) as points (11,884)	residences, castles, churches, glasshouses bridges, walls, fountains, gates, piers
Scheduled monument polygons* (8185)	Roman remains, standing stones, war memorials
Garden/landscape polygons* (391)	castle/stately home grounds, parks, cemeteries

(*shapes show approximate geographic size/extent).

#### 2.1.2 Deprivation data

For the area-level analysis our common spatial framework for local area characteristics was data zones. Data zones (*n* = 6976) are small area units created for collecting and reporting statistics about the Scottish population. They are broadly socially homogeneous and consistent in population size; however, their geographical size varies according to level of urbanicity/rurality (e.g., rural data zones are geographically larger as populations are more dispersed). Data zones have a median area size of 0.2 km^2^s (km²) and median population size of around 750 residents. We obtained data zone boundaries (2011) (Scottish Government, 2023) and linked these with the Scottish Index of Multiple Deprivation (SIMD) 2016 (income domain) score and population estimates to describe data zone population and socio-economic situation (Public Health Scotland, 2022). The SIMD income score is based on numbers of claimants for a range of welfare benefits e.g., Income Support, Jobseekers Allowance, Tax Credits etc. Descriptive statistics of SIMD Income quintiles (Q) (Q1 = most deprived) (including total, mean population/size per quintile) is included within supplemental Table 1.

#### 2.1.3 Area-level data linkage and analysis

We spatially joined data zone boundaries to heritage data (points or polygon centroids/centre points) using Geographic Information Systems software (ArcGIS Pro v3.0.3). We calculated a count of each heritage site in each data zone. Additionally, we used IBM SPSS Statistics v28 to calculate sites per 1000 population and per 0.5km² area to account for variation in data zone population and area when assessing distribution (see supplemental Table 1 for further information on variation). To explore whether area-level exposure to heritage sites varied by deprivation across Scotland, we compared data zone-level mean numbers of sites, and population- and area-weighted mean densities, across income deprivation quintiles, using Analysis of Variance.

### 2.2 Individual-level exposure to heritage (SPACES children)

To calculate individual-level exposure to heritage amongst SPACES children (i.e., ‘encounters’), we obtained data from (2.1) SPACES; (2.2) created exposure measures for GPS defined ‘activity spaces’ and compared these measures across income deprivation quintiles (see detail below).

#### 2.2.1 SPACES study participants

We used data from participants in the SPACES study (MRC/CSO SPHSU, 2023). The aim of which was to explore the environmental determinants of physical activity by conducting a large-scale, nationally representative, accelerometer and GPS observational study (McCrorie, Mitchell et al., 2018). The dataset collected detailed mobility data for children in Scotland linked to geocoded home and school addresses (during school term times, between May 2015 and May 2016). Participants were recruited from sweep 8 (interviews in 2014/2015) of the Growing Up in Scotland study, a nationally representative longitudinal cohort study originating in 2005. Each child was assigned an income deprivation quintile value (between Q1 (most deprived) and Q5 (least deprived)) based on their home location.

#### 2.2.2 SPACES children's heritage exposure

To quantify exposure, we created buffers from GPS collected via SPACES (*n* = 688 children returned GPS data). Within the study, children were given a GPS device (Qstarz BT-Q1000XT; Qstarz International Co., Ltd, Taiwan) and asked to wear it over eight consecutive days during waking hours. The device recorded the child's point location every ten seconds. We classified the presence of at least one GPS point as exposure, so that any type of ‘encounter’ could be captured, including passing by a historic building/green space. Using the ‘sf’ package in R (E. 2018, Dowle and Srinivasan, 2023), we created 10 m buffers around each point location. Buffers around points were merged to create a single ‘GPS buffer ’ representing the extent of each child's movements (i.e., their ‘activity space’). The 10 m threshold reflects the accuracy of GPS receivers; 78.7 % of GPS locations fall within 10 m of the actual location across travel modes and environments (Schipperijn, Kerr et al., 2014). We used the ‘Spatial Join’ (intersect) tool to link the heritage site points/polygons to GPS buffers and calculated a count of each heritage feature in each GPS buffer. To explore if children's heritage exposure within ‘GPS buffers’ varied by deprivation, we used Analysis of Variance.

## 3 Results

**Fig. 1:**
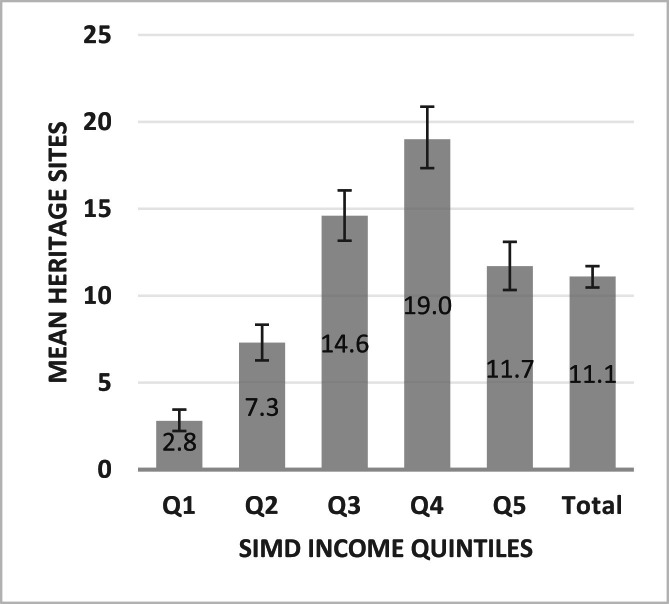
Area-level ‘opportunities’ - mean heritage sites by deprivation quintile.

Heritage exposure across Scotland varied by deprivation (descriptive information on quintile characteristics and heritage site numbers available within supplementary Table 1). Table 2 and Fig. 1 show mean heritage sites, and Confidence Intervals (CI), at area-level (data zones) by deprivation quintile. Mean number of sites varied by deprivation (*p* < 0.001), with a non-linear pattern across quintiles. Mean numbers increased from Q1 (2.8 (CI:2.2–3.5)) to Q4 (19.0 (CI:17.3–20.9)) (six-fold difference) and decreased within Q5 (11.7 (CI:10.3–13.1)). In post-hoc tests, mean numbers differed between each/every quintile (p-values <0.001 to 0.032). Population-/area-weighted densities showed similar patterns to non-weighted mean numbers of sites by deprivation (see supplementary Table 3), suggesting variation in means was not a result of variation in data zone population/area.

**Table 2:** Area-level ‘opportunities - mean heritage sites by deprivation quintile.

	**Area-level**
**Deprivation**	**Mean sites (95 % CIs)**
**1 (most deprived)**	2.8 (2.2–3.5)
**2**	7.3 (6.3–8.3)
**3**	14.6 (13.2–16.1)
**4**	19.0 (17.3–20.9)
**5 (least deprived)**	11.7 (10.3–13.1)
	
**Total (Scotland)**	11.1 (10.5–11.7)
**ANOVA**	*P* < 0.001, *F* = 83.92

(CIs, Confidence Intervals : upper-lower).

**Fig. 2:**
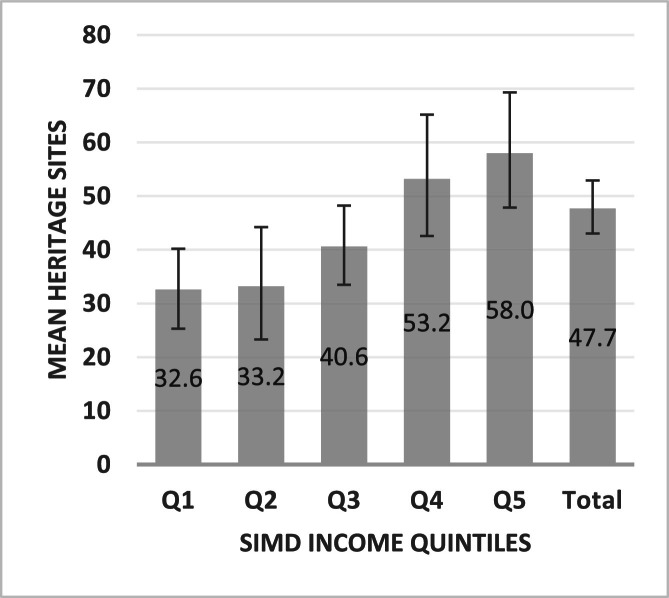
Individual-level ‘encounters’ - mean heritage sites by deprivation quintile.

Table 3 and Fig. 2 show mean number of sites within GPS buffers (individual-level) by deprivation quintile. Mean numbers varied by deprivation (*p* = 0.003) and showed a linear pattern across quintiles, with around a two-fold increase between the most and least deprived areas. Mean numbers in GPS buffers increased from Q1 (32.6 (CI:25.3–40.2)) to Q5 (58.0 (CI:47.9–69.3)); in post-hoc tests only Q1 and Q5 differed (*p* = 0.02). See Fig. 2 for graph.

**Table 3:** Individual-level ‘encounters’ - mean heritage sites by deprivation quintile.

	**Individual-level**
**Deprivation**	**Mean sites (95 % CIs)**
**1 (most deprived)**	32.6 (25.3–40.2)
**2**	33.2 (23.3–44.2)
**3**	40.6 (33.5–48.2)
**4**	53.2 (42.6–65.2)
**5 (least deprived)**	58.0 (47.9–69.3)
	
**Total (Scotland)**	47.7 (43.0–52.9)
**ANOVA**	*P* = 0.003, *F* = 3.98

(CIs, Confidence Intervals : upper-lower).

## 4 Discussion

This is the first study to explore the spatial distribution of heritage sites across areas of varying deprivation in Scotland, and to quantify individual day-to-day exposure to heritage sites using GPS data. Our findings indicated that at both area- and individual-level the most deprived areas had the lowest heritage exposure. Q1 areas had the smallest mean numbers of heritage sites/densities (i.e., fewer ‘opportunities’ for contact with heritage), and children living in Q1 areas had the lowest exposure (i.e., fewer ‘encounters’ with heritage). Despite some similarity in findings for both measures, the extent of inequality varied, that is, a starker difference at area-level (i.e., Q5 six-fold greater exposure than Q1) was seen than at individual-level (i.e., Q4 two-fold greater exposure than Q1). Irrespective of how inequality occurred, it could hinder opportunities to access heritage for some.

Our findings on the socio-spatial distribution of heritage in Scotland correspond to those for England (Macdonald, Nicholls et al., 2023). Akin to Macdonald et al.’s findings for LSOA ‘neighbourhoods’ in England, the most deprived data zone ‘neighbourhoods’ in Scotland had the lowest population-weighted densities of heritage sites (and area-weighted densities), while the second least and least deprived (Q4 and Q5) neighbourhoods had the highest. The rationale for this may be, firstly, that heritage can ‘create’ wealth. More heritage sites in affluent areas in Scotland (and England) could simply be attributed to heritage raising overall area wealth with, for example, listed buildings and residences in conservation areas more likely to sell for higher prices (Belford and Walker, 2021). Secondly, it may be that heritage ‘selects for’ wealth, in its capacity to generate monetary value by effectively exploiting and promoting heritage assets. Areas with greater concentrations of heritage may experience expanded economic development due to re-purposing of heritage assets for business, commerce, or tourism (Historic England, 2020), and, thus in turn, enhance local economies. This suggests a need to prioritise investment in heritage sites that are in, or close to, areas of deprivation. This approach could foster engagement/use of sites amongst local people, and stimulate local heritage site procurement and employment, as a means to provide potential economic (and well-being) benefits within areas most in need.

Regarding our individual-level analysis, using SPACES children's GPS data, our findings correspond to those of the area-level analysis to some extent; children in the most deprived areas had the lowest heritage exposure. Such findings could be partly explained by variation in GPS buffer area, and on average smaller GPS-defined distances travelled for children in Q1 and greater distances for those in Q5 (see supplemental Table 2). In other words, children in the least deprived areas travelled further and thus accumulated contact with a higher number of heritage sites in their daily lives. Smaller GPS-buffers for children in deprived areas may, in part, be attributed to shorter home-to-school distances (Zhu and Lee, 2008), with greater geographical availability of primary schools seen in poorer areas (Macintyre, Macdonald et al., 2008; Olsen, Caryl et al., 2023). Regarding the difference in the extent of inequality at area- and individual-level, it appears that disparities are somewhat mitigated when individual mobility is considered. This suggests that children in more deprived areas compensate, to some degree, for their lack of heritage exposure at home during their day-to-day movements. Our finding that Q4 areas had the highest number of opportunities, while children in Q5 had the highest number of encounters, suggests that these children also compensated for fewer heritage sites proximal to home through their daily mobility (or this could indicate greater repeated encounters to the same sites). Despite accounting for individual mobility, we found inequalities in exposure persisted, thus opportunities for individuals to access heritage remain unequal to the detriment of those in poorer areas.

## 5 Policy implications

Our findings contribute to further understanding of Scotland's geographical availability of heritage sites, children's day-to-day contact with heritage, and socio-spatial inequalities therein. Findings are relevant to the Scottish Government's National Planning Framework (Scottish Government, 2020) with a focus on heritage as a national asset supporting current and future generations’ health and well-being, and to Scotland's new historic environment strategy emphasising improving heritage access for all (HES, 2023). The framework acknowledges that individuals in Scotland experience varying life chances and opportunities, which are, in part, influenced by where they live; our evidence on unequal access to cultural assets, such as heritage, fits this narrative. The primary policy-related implication from our findings underscores the importance of improving geographical accessibility to heritage. In particular, for those within deprived ‘low exposure’ areas who typically may not travel further to additional destinations. Local living policies value geographical accessibility to amenities, public transport, public, open, and natural spaces (Scottish Government, 2020), but do not explicitly address heritage, perhaps as these sites cannot be newly built into neighbourhoods. Improved local public transport within deprived areas could facilitate visits to heritage, however, financial barriers to access must also be addressed. For example, providing subsidised transport and free entry to heritage sites for those on lower incomes. Historic Environment Scotland work with outside partners (e.g., charities for the elderly) to provide bespoke free entry events for some, however, there appears to be no geographically targeted schemes aimed at areas of deprivation. We need discourse on optimal approaches ‘to bring heritage’ to these areas, which enhance not only opportunities to visit heritage but also interest in heritage. Bringing heritage to lower exposure/deprived areas could include innovative ‘creative outreach’ strategies, for instance, sharing heritage artefacts or images with communities to create interest; an approach proven successful for museums (Pathways to Wellbeing, 2022). Investment in programmes to increase awareness of local heritage, such as, establishing heritage trails and providing informative signage to guide residents or visitors to heritage features that they may be unaware of. Education outreach programmes could be used to increase young people's heritage awareness, for example, school field trips to sites. One England-based study assessed over 900 secondary school students’ value of heritage through field trips with GPS-enabled cameras to capture local historic buildings (Bradley, Coombes et al., 2011). Nearly 60 % of students found local historic buildings to be important to them and made them feel proud of their area, however, students in deprived areas expressed lower place attachment and noted fewer historic buildings locally. Bringing heritage into the awareness of young people in deprived neighbourhoods could be a way to create a positive sense of place and belonging for them (Blamire, Rees et al., 2022). Neighbourhood characteristics are shown to influence a young person's development; providing stimulation and opportunities to learn, explore, play, and socialise (Villanueva, Badland et al., 2016); heritage could serve as an additional environmental characteristic that enriches young people's lives.

## 6 Strengths and limitations

Our study was strengthened by inclusion of heritage exposure at area- and individual-level. We go beyond studies focussing solely on exposure inferred by availability to include ‘real-life’ contact with heritage, using objective data from GPS devices worn over multiple days. We use a novel application of GPS data, and a robust methodology, to quantify heritage exposure for SPACES children Scotland-wide. Additionally, we collaborated with Historic Environment Scotland throughout the research process to optimize the generation of valuable research evidence. Regarding limitations, we include designated sites only, and non-designated sites, that is, historic assets that do not have formal or legal designations attached to them (but may still hold local value), are not included as such spatial data is unavailable. We cannot assume that all scheduled monuments are completely visible, as some may be archaeological sites/ruins that are partly underground. We did not measure repeated encounters with the same sites (unfeasible with GPS buffers used) which could appear to amplify the measure of exposure for some. We acknowledge that spatial exposure is only one aspect of accessibility; barriers beyond geographic access exist, whether financial (e.g., inability to pay for admittance fees/transport), practical (e.g., lack of information on heritage sites), emotional (e.g., inability to see value in heritage) etc. (Whitaker, 2016). We recognise that in our study we do not differentiate between type of heritage encounter, such as passing by a site versus engaging in a purposeful visit. Children within more or less deprived areas may experience variation in type of encounter; some experiences may be more or less meaningful than others. Future research could employ similar GPS tracking techniques, alongside more qualitative data collection, to obtain detailed information about where/how individuals encounter heritage. Additional insights will be gained by examining if these various types of encounters are linked to health and well-being. Regarding barriers to accessibility, we did not explore the value of heritage to young people. Young people's perception of heritage as ‘irrelevant’ remains a significant obstacle for heritage organisations (Heritage Lottery Fund, 2015). From our study, despite quantifying contact with heritage, we know little of engagement with it. Nonetheless, for there to be meaningful contact the presence/reachability of heritage is key, and further research is needed to explore the value of children's daily interactions with heritage. Finally, we acknowledge that our conclusions are Scotland-specific and may not be internationally applicable, however, our methodology could be utilised in other regions with available heritage data.

## 7 Conclusion

Our findings demonstrated that the most deprived areas in Scotland had the lowest numbers and densities of heritage sites, and, through a novel application of GPS data, we found that children living in the most deprived areas had the lowest exposure to heritage. For those in deprived areas (compared to their less deprived counterparts), area-based analysis indicated residents had fewer ‘opportunities’ to encounter heritage sites locally, while individual-based analysis confirmed that residents had fewer ‘real-life’ encounters with heritage. Individual-level inequalities were smaller in magnitude than those at area-level, suggesting that children in poorer areas compensated for fewer proximal sites at home during their everyday movements. Although there was a reduction in inequalities at individual-level, inequality persisted; opportunities to access heritage continue to remain unequal to the detriment of those in more deprived areas. We consulted with Historic Environment Scotland throughout the research process to maximise creation of valuable research evidence, and we believe our findings are relevant to heritage-related policy, emphasising the need to enhance geographical access to heritage sites in low exposure/more deprived areas. This may include prioritising economic investment in heritage sites in/proximal to areas of deprivation; for those on lower incomes providing subsidised travel to high exposure areas and free site entry; and ‘bringing’ heritage into the awareness of local communities and young people via cultural and educational outreach activities.

## Ethical approval

SPACES data collection took place between May 2015 and May 2016 and ethical approval was provided by the College of Social Sciences, University of Glasgow (CSS ref: 400,140,067).

## Funding statement

LM, FC, and RM are employed by the MRC/CSO Social and Public Health Sciences Unit, University of Glasgow, and supported by the 10.13039/501100000265Medical Research Council [grant number MC_UU_00022/4] and 10.13039/501100000589Chief Scientist Office [grant number SPHSU19]. FC is supported by an MRC Skills Development Fellowship [MR/T027789/1].

## Data sharing statement

For further information, please refer to the SPACES study data sharing portal at http://spaces.sphsu.mrc.ac.uk/.

## CRediT authorship contribution statement

**Laura Macdonald:** Conceptualization, Methodology, Formal analysis, Writing – original draft. **Fiona Caryl:** Conceptualization, Writing – review & editing. **Richard Mitchell:** Conceptualization, Funding acquisition, Writing – review & editing.

## Declaration of competing interest

The authors declare the following financial interests/personal relationships which may be considered as potential competing interests: Richard Mitchell reports financial support was provided by UKRI Medical Research Council. Richard Mitchell reports financial support was provided by Chief Scientist Office. If there are other authors, they declare that they have no known competing financial interests or personal relationships that could have appeared to influence the work reported in this paper.

## Appendix Supplementary materials

Image, application 1
