# Supplementary material for: Inequalities in geographical distribution of heritage in Scotland, investigating spatial exposure to heritage sites through area-based and individual-based (GPS) measurement
Source: Wellbeing Space Soc. 2024 Dec;7:100211. doi: 10.1016/j.wss.2024.100211 (PMC11649978; doi:10.1016/j.wss.2024.100211)
Supplement: Supplementary file 1 [file mmc1.docx]

**Supplementary tables**

**Supplementary table 1. SIMD income deprivation quintile descriptive – population, area, heritage sites**

| **Deprivation** | **Population**  **(data zone mean)** | **Area km²**  **(data zone mean)** | | **Number of heritage sites** | |  |
| --- | --- | --- | --- | --- | --- | --- |
| **1 (most deprived)** | 1,052,709 (760.8) | | 347.4 (0.4) | | 4,038 | |
| **2** | 1,056,540 (752.1) | | 3875.3 (2.7) | | 10,278 | |
| **3** | 1,083,412 (784.3) | | 23,460.6 (18.1) | | 19,065 | |
| **4** | 1,141,919 (814.9) | | 37,576.2 (26.2) | | 26,906 | |
| **5 (least deprived)** | 1,131,420 (805.6) | | 12,677.6 (8.6) | | 16,484 | |
| **Scotland** | 5,466,000 (783.5) | | 77,937.1 (11.2) | | 76,771 | |

(Quintiles 1,2,4,5 contain 1,395 data zones each, Q3 contains 1,396)

**Supplementary table 2. SPACES children – mean GPS buffer area (km²) by SIMD income deprivation quintile**

| **Deprivation** | **Mean buffer area (km²)** |
| --- | --- |
| **1 (most deprived)** | 0.34 |
| **2** | 0.38 |
| **3** | 0.39 |
| **4** | 0.45 |
| **5 (least deprived)** | 0.43 |
| **Total (SPACES children)** | 0.41 |

(GPS buffer count Q1=64, Q2=90, Q3=148, Q4=178, Q5=208, Total=688)

**Supplementary table 3. Population-/area-weighted mean densities of heritage sites (data-zone level), by SIMD income deprivation quintile.** *(CIs, Confidence Intervals: upper-lower)*

|  | Data zone-level | |
| --- | --- | --- |
|  | **Population-weighted** | **Area-weighted** |
| Deprivation | **Mean sites (95% CIs)** | **Mean sites (95% CIs)** |
| 1 (most deprived) | 3.6 (2.9-4.5) | 7.2 (5.9-8.5) |
| 2 | 9.6 (8.2-11.0) | 16.8 (13.9-19.6) |
| 3 | 19.4 (17.4-21.6) | 20.3 (16.8-24.2) |
| 4 | 24.2 (21.8-26.6) | 18.9 (15.2-22.9) |
| 5 (least deprived) | 14.3 (12.6-15.8) | 23.9 (18.6-28.9) |
| Total (Scotland) | 14.2 (13.4-15.0) | 17.4 (15.8-18.9) |
| ANOVA | P<0.001, F=80.60 | P<0.001, F=11.66 |
